# The Sound of Health

**DOI:** 10.3389/fimmu.2014.00351

**Published:** 2014-07-21

**Authors:** Giamila Fantuzzi

**Affiliations:** ^1^Department of Kinesiology and Nutrition, University of Illinois at Chicago, Chicago, IL, USA

**Keywords:** adiponectin, danger signals, stress, physiological, philosophy, metabolism, osteocalcin, Klotho

## The Silence of Health

“*Health is life lived in the silence of the organs*” wrote the French surgeon René Leriche in 1936, a concept later adopted and further developed by the physician and philosopher Georges Canguilhem (1). Albeit certainly resonant with the experience of health defined as the state of well being perceived by an individual, this concept is less valid when health is evaluated in terms of physiological homeostasis. Here, I will introduce the notion that healthy cells are actively involved in signaling for the presence of health, as contrasted to the widely accepted concept that stressed cells send out messages indicating lack of health. I propose that, although true at the level of human beings feeling the state of their own health, Diderot’s view that “*When we are well, no part of the body informs us of its existence*” (2) does not apply to the physiology of cells, tissues, organs, and the body as a whole.

## Messages of Distress

It is a widely accepted notion that cells subjected to stress – be it of a physical, chemical, metabolic, infectious or other origin – react through a coordinated response that leads to production of signals communicating the presence of stress and danger to other cells. These pathways have been magistrally defined and delineated by Chovatiya and Medzhitov in a recent article (3). The end result of the elaboration and propagation of signals of distress is the molecular, functional and structural reconfiguration of cells and tissues to face conditions of lack of health and eventually promote the return to homeostasis if and when the injurious stimulus is removed, or to chronicization if the stimulus persists. Examples of such responses include increase in vascular permeability and induction of inflammatory infiltrates, development of insulin resistance, degradation of bone and cartilage, disruption of the gut epithelial barrier, altered neuronal function, inappropriately increased or decreased cell proliferation or cell death, development of fibrosis, and many more.

The notion of the body responding to lack of health by generating signals of distress has been extremely productive in furthering our understanding of mechanisms of disease and developing therapeutic strategies. However, although not explicitly stated, this view implies that, whereas disease makes a lot of noise, health is mostly silent.

## Messages of Health

But silent health is not. I propose that availability of messages of health is at least as important in determining the state of an organism as is the intensity of messages of distress. Although much has been written to define and classify these latter, a comparable effort is lacking for messages of health. This situation perhaps results from the unstated, and for the most part unconscious, belief of scientists that, again with Canguilhem, “*If health is life in the silence of the organs, then, strictly speaking, there is no science of health. Health is organic innocence. It must be lost, like all innocence, so that knowledge may be possible*” (1). Though conceivable at the philosophical level, physiological health does not have much to do with innocence but is rather the result of highly sophisticated, coordinated, active biological strategizing that can and should be studied as part of a science of health. I believe we can approach biomedical research from the point of view of studying what improves health in addition to, but not as a substitute of, investigating what causes disease. Identifying and understanding the nature and regulation of messages of health would be central to this enterprise, part of the larger task, highlighted by Chovatiya and Medzhitov, aimed at defining what constitutes homeostasis at the tissue and organ level (3).

I define messages of health as molecules produced and released by healthy, unstressed cells and whose presence contributes to support a healthy organism. Levels and/or activity of messages of health are decreased in response to stress and this reduction contributes to pathogenesis, thus providing a mirror image to the behavior of messages of distress. This definition is based on the properties of the adipokine adiponectin, a protein produced and released by adipocytes (4, 5). The small adipocytes of a lean, insulin-sensitive individual without cardiovascular disease, particularly if this person is a woman, produce high levels of adiponectin, high enough to reach circulating levels in the tens of micrograms per milliliter range. Thus, healthy adipocytes spend a considerable amount of energy (in the form of ATP) and matter (in the form of amino acids) to produce tremendous amounts of adiponectin. Signaling by this adipokine in a variety of organs is critical in maintaining metabolic and vascular health and providing an overall anti-inflammatory tone (4, 5). According to the above definition, stress should lead to reduced levels or activity of messages of health; this can occur secondary to decreased gene expression or through alterations in the processes of post-transcriptional and/or post-translational modifications, reduced release into the extra-cellular space as well as diminished bioavailability and bioactivity. Indeed, exposure to a variety of stresses reduces adiponectin production, both *in vitro* and *in vivo* (4, 5). In obesity, as adipose tissue expands and adipocytes become larger and as glucose intolerance and insulin resistance develop, production of adiponectin significantly declines. Moreover, obesity is not only associated with reduced adiponectin mRNA and protein levels but also with alterations in its multimerization properties, transport across endothelium as well as perhaps development of adiponectin resistance (4–7). Evidence indicates that the decreased adiponectin levels and activity that accompany obesity, diabetes and cardiovascular disease can be both cause and effect of these pathologies (4, 5). Moreover, polymorphisms in the adiponectin gene that affect rate of production, release and ability to multimerize are associated with risk of developing metabolic disease (4–7). Thus, adiponectin’s production and activity are maximal under conditions of health and are reduced by stress, with this reduction causally contributing to the pathogenesis of metabolic diseases.

Is adiponectin’s biology unique? Can the definition of messages of health be applied to other molecules? Potential candidates exist, though they have not been as extensively studied as adiponectin and therefore not as many facets of their biology are currently known. Osteocalcin is a small protein produced and released by osteoblasts; it is stored in the bone matrix and is released into the circulation as bone gets physiologically remodeled, acting as an endocrine hormone. Osteocalcin exerts beneficial effects on systemic glucose and lipid metabolism, inhibits inflammation, and supports production of testosterone (8, 9). In humans, circulating levels of osteocalcin are negatively correlated with markers of the metabolic syndrome and diabetes, while limited evidence supports the association between genetic variants in osteocalcin and its receptors and dysregulated glucose metabolism (8, 9). Another candidate message of health is Klotho, aptly named after the Greek Fate spinning the thread of life (10). Klotho (α Klotho, to be precise) is mainly produced by the kidney and exists as both a cell-associated and a soluble protein, generated by either alternative splicing or ectodomain shedding of the membrane-bound form. Soluble Klotho has a variety of salutary effects, including regulation of insulin release, inhibition of insulin growth factor signaling, protection from cell senescence, modulation of renal function and prevention of renal fibrosis, maintenance of vascular health and downregulation of inflammatory responses (9, 11, 12). In keeping with the concept that messages of health are reduced by stress, levels of soluble Klotho are low in people with diabetes (13) and decline with age (14).

As these examples illustrate, messages of health are molecules that are optimally produced, released and bioactive in the absence of stress and that communicate the presence of health – or lack of distress – however one prefers to frame the concept. To be sure, flexibility is essential when conceptualizing a molecule as a message of health or distress, as crossover situations are not that rare and can occur in either direction. On the side of messages of health turned into signals of distress we find, for example, adiponectin levels being elevated in some chronic conditions and possibly contributing to disease pathogenesis (15), while on the opposite side of the coin one can mention, among several options, the pro-inflammatory cytokine TNFα having both beneficial and detrimental effects in the intestinal environment (16) and the alarmin HMGB1 promoting autophagy and maintaining telomere integrity (17). As always, context is crucial and categorizations need to be taken with a grain of salt. However, with these qualifications in mind, molecules that are for the most part produced and released by healthy cells and carry messages of health can be distinguished from those that are generally produced under conditions of distress and signal for lack of health. Even though we have a relatively good understanding of the molecular pathways that signal for the presence of distress, the concept of messages of health has not yet been formally proposed and therefore identification, characterization and categorization of these molecules is lagging behind. As a consequence, at present the sound of health is still a whisper, and a major challenge ahead will be to understand whether it consists of a simple melody or is instead a glorious symphony.

## The Sound of Silence

Despite being biologically unrelated, the notion of messages of health owes much to the concept of resolution of inflammation, defined as the “*coordinated and active process aimed at restoration of tissue integrity and function*” following an inflammatory insult (18). Here, the stress of inflammation induces production of mediators that actively suppress the inflammatory response, a process that can potentially be exploited through interventions aimed at increasing synthesis or activity of mediators of resolution, as opposed to the traditional approach of suppressing production of pro-inflammatory factors. As indicated by the authors, “*to transfer concepts of resolution from bench to bedside requires a shift in emphasis from inhibitory therapy to replacement therapy*, i.e., *from antagonism to agonism*” (18). Messages of health are, by definition, inhibited by stress and therefore do not overlap with mediators of resolution. However, the concept of promoting production and activity of messages of health instead of inhibiting messages of distress, of using agonist rather than antagonist approaches, is consistent with the strategies proposed in the field of resolution. A science of health could thus lead to identification and characterization of additional messages of health and to design and implementation of strategies aimed at actively promoting health.

It is not simply the absence of signals of distress that allows for Leriche’s “silence of the organs,” but also the presence of messages of health, of molecules that form the biological notes of that awesome sound of silence we call health.

## Conflict of Interest Statement

The author declares that the research was conducted in the absence of any commercial or financial relationships that could be construed as a potential conflict of interest.
